# Fitness costs of key point mutations that underlie acaricide target‐site resistance in the two‐spotted spider mite *Tetranychus urticae*

**DOI:** 10.1111/eva.12643

**Published:** 2018-05-20

**Authors:** Sabina Bajda, Maria Riga, Nicky Wybouw, Stavrini Papadaki, Eleni Ouranou, Seyedeh Masoumeh Fotoukkiaii, John Vontas, Thomas Van Leeuwen

**Affiliations:** ^1^ Laboratory of Agrozoology Department of Plants and Crops Faculty of Bioscience Engineering Ghent University Ghent Belgium; ^2^ Institute for Biodiversity and Ecosystem Dynamics University of Amsterdam Amsterdam the Netherlands; ^3^ Department of Biology University of Crete Heraklion, Crete Greece; ^4^ Institute of Molecular Biology & Biotechnology Foundation for Research & Technology Hellas Heraklion, Crete Greece; ^5^ Laboratory of Pesticide Science Department of Crop Science Agricultural University of Athens Athens Greece

**Keywords:** acaricide resistance management, chitin synthase 1, cytochrome b, fitness cost, glutamate‐gated chloride channels, target‐site resistance, *Tetranychus urticae*, voltage‐gated sodium channels

## Abstract

The frequency of insecticide/acaricide target‐site resistance is increasing in arthropod pest populations and is typically underpinned by single point mutations that affect the binding strength between the insecticide/acaricide and its target‐site. Theory predicts that although resistance mutations clearly have advantageous effects under the selection pressure of the insecticide/acaricide, they might convey negative pleiotropic effects on other aspects of fitness. If such fitness costs are in place, target‐site resistance is thus likely to disappear in the absence of insecticide/acaricide treatment, a process that would counteract the spread of resistance in agricultural crops. Hence, there is a great need to reliably quantify the various potential pleiotropic effects of target‐site resistance point mutations on arthropod fitness. Here, we used near‐isogenic lines of the spider mite pest *Tetranychus urticae* that carry well‐characterized acaricide target‐site resistance mutations to quantify potential fitness costs. Specifically, we analyzed P262T in the mitochondrial cytochrome b, the combined G314D and G326E substitutions in the glutamate‐gated chloride channels, L1024V in the voltage‐gated sodium channel, and I1017F in chitin synthase 1. Five fertility life table parameters and nine single‐generation life‐history traits were quantified and compared across a total of 15 mite lines. In addition, we monitored the temporal resistance level dynamics of populations with different starting frequency levels of the chitin synthase resistant allele to further support our findings. Three target‐site resistance mutations, I1017F and the co‐occurring G314D and G326E mutations, were shown to significantly and consistently alter certain fitness parameters in *T. urticae*. The other two mutations (P262T and L1024V) did not result in any consistent change in a fitness parameter analyzed in our study. Our findings are discussed in the context of the global spread of *T. urticae* pesticide resistance and integrated pest management.

## 1 INTRODUCTION

Control of arthropod pests in agriculture remains heavily dependent on the application of insecticides and acaricides, a practice that has resulted in the current widespread occurrence of resistant populations of more than 500 arthropod species (Sparks & Nauen, 2015). Resistance of an arthropod population to a pesticide is typically defined as the heritable decrease in the susceptibility to the sprayed pesticide that leads to inadequate field control (http://www.irac-online.org/about/resistance/). Adaptation to xenobiotics, including insecticides and acaricides, is mainly attributed to an elevated activity of detoxification enzymes (toxicokinetic change) and/or a reduced binding strength of the xenobiotic to its target‐site (toxicodynamic change). Within the latter mechanism, nonsynonymous point mutations in the sequence coding for the pesticide target‐site are most often reported (Feyereisen, Dermauw, & Van Leeuwen, 2015). Theory predicts that de novo point mutations in essential target genes can convey pleiotropic effects, meaning they could affect other phenotypic traits in addition to pesticide resistance (ffrench‐Constant & Bass, 2017; Crow, 1957; Fisher, 1999). Indeed, these point mutations may, for instance, impose a structural constraint and put the conserved protein function and therefore the arthropod’s fitness at a disadvantage. Pesticide resistance could thus result in fitness costs in populations that live in a pesticide‐free environment. As a consequence, alleviating pesticide use could result in a lower frequency of target‐site resistance alleles and, in turn, a lower resistance level of the population (Crow, 1957; Georghiou & Taylor, 1977). Target‐site resistance alleles can however be maintained in pest populations through several mechanisms. The pleiotropic fitness costs may be negligible or compensated via additional mutations. These additional mutations, or fitness modifiers, restore fitness to its original level and have been well studied in antibiotic‐resistant bacteria (Björkman, Nagaev, Berg, Hughes, & Andersson, 2000; Comas et al., 2012; Gagneux et al., 2006; Levin, Perrot, & Walker, 2000). A notable example of a fitness modifier within the Arthropoda phylum has been found in the Australian blow fly, *Lucilia cuprina,* where the scalloped wings gene *ScI* is a likely candidate for the fitness and wing asymmetry modifier in diazinon‐resistant flies (Davies et al., 1996). Alternatively, the resistance locus can be physically linked to a locus that confers a selective advantage and thus persists by mere linkage disequilibrium.

Experimental verification whether the mutations that underlie insecticide/acaricide resistance indeed carry fitness costs, typically relies on two methodologies (Roush & Daly, 1990). The first method investigates various single‐generation life‐history parameters. However, here the cost of a causal resistance mutation can easily be missed in experimental designs that only look at a specific fitness component. Indeed, population growth depends on a multitude of interdependent life‐history traits (LHTs) and their cumulative effect on population dynamics can only be estimated via complex parameters such as fertility life table parameters (LTPs; Roush & McKenzie, 1987). The second approach, often referred to as a “population cage” experiment because of its analogy to the traditional cage studies investigating *Drosophila melanogaster* genetics, analyzes fitness differences by placing resistant and susceptible genotypes in direct competition (Moore, 1952). These intergenotype competition experiments are run in the absence of pesticide exposure and allow tracking the frequency of resistance alleles (or the resistance phenotype itself) over multiple generations.

Excluding a number of studies that have focused on mosquitoes [*Culex pipiens quinquefasciatus* (Berticat, Boquien, Raymond, & Chevillon, 2002; Berticat et al., 2008; Gazave, Chevillon, Lenormand, Marquine, & Raymond, 2001), *Aedes aegypti* (Brito et al., 2013), and *Anopheles gambiae* (Diop et al., 2015)], the Australian blow fly *L. cuprina* (McKenzie, 1990, 1994), and the peach aphid *Myzus persicae* (Foster, Denholm, & Devonshire, 2000), the majority of previous work that assesses pesticide resistance‐related fitness costs in arthropods suffers from multiple design weaknesses [see also reviews by ffrench‐Constant and Bass (2017) and Kliot and Ghanim (2012)]. A common design flaw is the evaluation of genetically unrelated populations in the experimental setup. The different genetic background and adaptive variations in life‐history traits across such populations hamper any reliable claim of a causal effect of the point mutation of interest to the observed differences in population growth dynamics (Raymond, Wright, & Bonsall, 2011; The Anopheles gambiae 1000 Genomes Consortium, 2017; Varzandeh, Bruce, & Decker, 1954). An elegant solution to overcome this experimental limitation is to backcross the target‐site mutation of interest into a susceptible genomic background over multiple generations, hereby generating near‐isogenic lines. This procedure maximizes the chance that the observed difference in population growth is caused by the target‐site mutation under investigation (Bajda et al., 2017; Brito et al., 2013; Riga et al., 2017). Unfortunately, the biological characteristics of many insect and mite pests render the generation of near‐isogenic lines extremely difficult and time‐consuming.

The two‐spotted spider mite, *Tetranychus urticae* (Chelicerata: Acari: Tetranychidae), is one of the most notorious agricultural arthropod pests worldwide. *T. urticae* infests a wide range of different plant species (>1,000), of which many are economically important crops (Jeppson, Keifer, & Baker, 1975; Migeon & Dorkeld, 2006). Control of *T. urticae* populations is mainly accomplished by acaricide application and has led to a record number of populations resistant to pesticides with varying modes of action (Van Leeuwen & Dermauw, 2016; Van Leeuwen, Vontas, Tsagkarakou, Dermauw, & Tirry, 2010). Acaricide target‐site resistance has been widely reported in *T. urticae* field populations, and, facilitated by available genomic resources, a number of point mutations conferring target‐site resistance have been uncovered. Recently, Riga et al. (2017) investigated to what extent point mutations in a set of highly conserved acaricide target‐sites underlie the high resistance levels observed in *T. urticae* field populations. Nine point mutations within four conserved genes were introduced in a susceptible genomic background by marker‐assisted backcrossing. The study revealed that the presence of target‐site mutations in the highly conserved chitin synthase 1 (CHS1; I1017F), voltage‐gated sodium channel (VGSC; L1024V, F1538I), and mitochondrial cytochrome b (cytb; S141F + G126S, P262T) is sufficient to attain very high resistance levels (without the need of additive effects conferred by other genetic mechanisms). In contrast, the presence of the mutations in glutamate‐gated chloride channels 1 and 3 (GluCl1; G314D and GluCl3; G326E) results in much lower resistance levels compared to those reported in resistant field populations (Riga et al., 2017), although in vitro work established that the G326E mutation in single receptor genes abolishes the antagonistic interactions of macrocyclic lactones (Mermans, Dermauw, Geibel, & Van Leeuwen, 2017).

In this study, we further take advantage of this set of isogenic lines to evaluate the potential fitness costs associated with five target‐site resistance mutations. Specifically, we focused on P262T in the mitochondrial *cytb* associated with bifenazate resistance (Van Leeuwen et al., 2008), the combined effect of G314D and G326E in the *GluCl* channels associated with avermectin resistance (Dermauw et al., 2012; Kwon, Yoon, et al., 2010), L1024V in the *VGSC* associated with pyrethroid resistance (Kwon, Clark, et al., 2010), and I1017F in *CHS1* associated with resistance to etoxazole, clofentezine, and hexythiazox (Demaeght et al., 2014; Van Leeuwen et al., 2012). Five LTPs [the net reproductive rate (R0), intrinsic rate of increase (rm), mean generation time (*T*), the finite rate of increase (LM) and the doubling time (DT)], and nine single‐generation LHTs (immature stage survivorship [ISS], developmental time, sex ratio, adult longevity, daily and total fecundity, length of pre‐, post‐, and oviposition periods) were analyzed and compared across a total of 15 lines. In addition, we monitored the temporal resistance level dynamics of populations with different starting frequencies of the *CHS1* resistant allele to further support our findings. Our results indicated that three of the five studied target‐site mutations have a negative impact on *T. urticae* population growth, in absence of the respective acaricide. Our results help to understand and predict the occurrence and spread of *T. urticae* pesticide resistance within an integrated pest management context.

## 2 MATERIALS AND METHODS

### 2.1 Spider mite strains

A total of 15 *T. urticae* near‐isogenic lines were used in this study. The near‐isogenic lines were generated in a previous study using a marker‐assisted backcrossing technique (Bajda et al., 2017; Riga et al., 2017). Briefly, these were lines that carry introgressed nucleus‐encoded nonsynonymous mutations in *CHS1* gene (CHS1_R1‐R3), *VGSC* gene (VGSC_R2 and R3), *GluCl* genes (GluCl1+3_R1‐3) and their congenic susceptible controls (CHS1_C, VGSC_C1 and GluCl1+3_C, respectively) as well as lines carrying an introgressed mitochondrial‐encoded mutation in *cytb* gene (cytb_R1‐R3). The congenic susceptible control for the latter mutation was the susceptible Wasatch strain. An overview of these lines is shown in Table 1. All *T. urticae* strains were maintained on detached 3‐week‐old kidney bean leaves (*Phaseolus vulgaris* L.) on wet cotton wool inside lid‐covered plastic trays. Except when analyzing temporal etoxazole resistance dynamics, all mite cultures and experiments were kept under laboratory conditions at 25 ± 1°C, 60% relative humidity, and 16:8 hr light:dark photoperiod. No human subjects have participated in this project.

**Table 1:** Characteristics of the *Tetranychus urticae* near‐isogenic lines used in this study. The VGSC mutation was numbered according to *Musca domestica* numbering, whereas substitutions in *GluCl1, GluCl3, cytb*, and *CHS1* followed *T. urticae* numbering. IRAC mode of action group number is shown between brackets

Gene	Near‐isogenic line/susceptible strain^a^	Mutation	Target‐site resistance to
*CHS1*	CHS1_C	—	—
	CHS1_R1, R2, R3	I1017F	Mite growth inhibitors (10)
*Cytb*	Wasatch	—	—
	cytb_R1, R2, R3	P262T	Bifenazate (20A)
*VGSC*	VGSC _C1	—	—
	VGSC _R2, R3	L1024V	Pyrethroids (3A)
*GluCl1* and *GluCl3*	GluCl1+3_C,	—	—
	GluCl1+3_R1, R2, R3	G314D+G326E	Avermectins (6)

a Lines carrying mutation in *cytb* were compared to the susceptible strain Wasatch.

### 2.2 Total development time, sex ratio, and immature stage survivorship

From stock cultures of each of the backcrossed lines, four replicates of 100 adult female mites were randomly chosen and placed on a feeding arena (one 3‐week‐old bean leaf, lined with tissue paper to prevent escape). Mites were allowed to lay eggs for 4–5 hr and were subsequently moved to a new feeding arena for another 4–5 hr. The amount of eggs laid per plate was recorded for ISS calculations. Here, ISS was defined as the fraction of females reaching adulthood. On the eighth day after egg laying, mite development was followed every 12 hr. Eclosion was timed and adult individuals were sexed.

### 2.3 Oviposition and adult longevity

Thirty‐five single‐pair crosses were established per line by placing a female teliochrysalis with an adult male on a leaf disk of 3 cm^2^. Disk edges were lined with tissue paper to prevent mites from escaping. Once females reached adulthood, each pair was transferred daily to a fresh leaf disk. Males that did not survive for 2 days after their female partner reached adulthood were replaced. The ovipositional period was divided into three parts: preoviposition, oviposition, and postoviposition. The preoviposition period was determined as the time spanning between adult female emergence and the first egg and was estimated from observations taken every 12 hr. The oviposition period was defined as the time between the first and last day of egg laying. The postoviposition period was measured from the day when no more eggs were deposited for a given female, until her death. Oviposition and the subsequent postoviposition periods were monitored on a daily basis until the death of each female. Data from females that died due to experimental manipulation were excluded from further analysis.

### 2.4 Temporal dynamics of etoxazole resistance

Etoxazole resistance was evaluated monthly for a period of 8 months for three *T. urticae* populations originating from the cross CHS1_R2 × CHS1_C, with different initial proportions of etoxazole‐resistant and susceptible mites: R70: 70/30, R50: 50/50, and R30: 30/70. Two hundred adult females were placed as the founding population and maintained on potted nonsprayed bean plants in a Panasonic climate chamber (MLR‐352H‐PE), at 28°C with a photoperiod of 16:8 hr light:dark. To assess etoxazole resistance over time, larval bioassays were performed as previously described by Van Pottelberge, Khajehali, Van Leeuwen, and Tirry (2009). Briefly, 50 adult females were allowed to lay eggs for 5 hr on the upper side of 9 cm^2^ square‐cut bean leaf disks on wet cotton wool. Larvae were sprayed with 1 ml of etoxazole (100 mg/L) at 1 bar pressure with a Potter Spray Tower (Burkard Scientific, UK; 2 mg/cm^2^ aqueous deposit). A commercial formulation of etoxazole (Borneo, 120 g/L SC) was used in this study. The discriminating etoxazole concentration is based on previously obtained mortality data (Van Leeuwen et al., 2012). Mortality was assessed after 48 hr. Here, mites that displayed the same developmental stage as the water‐treated control were considered as unaffected. All mortalities obtained from control treatment were lower than 10%. At each time point, the resistance level of the three populations was assessed using three to four replicates of approximately 50 larvae on each.

### 2.5 Statistical analysis

ISS and sex ratio were analyzed with logistic regression using the *glm* function with a binomial error distribution (package *stats*).

Female longevity, total and daily fecundity, and duration of preoviposition, oviposition, and postoviposition periods were analyzed in a linear model using *lm* (package *stats*). To describe differences in developmental time (defined as the time required for ≥50% of individuals to reach adulthood), a linear mixed effect model was initially run (function *lme* from package *nlme*) where “line” was a fixed effect and the “time block” (representing the two feeding arenas upon which replicates were allowed to lay eggs) was considered as a random effect. As determined by the *anova* function from package *stats*, the random effect of time block was insignificant; hence, data were finally analyzed with a linear model using *lm,* where every plate was treated as an independent biological replicate.

Post hoc Dunnett’s test was subsequently carried out to perform multiple comparisons with the control line, using the *glht* function in the package *multcomp* (adjusted *p*‐value <0.05; Westfall, Tobias, Rom, Wolfinger, & Hochberg, 2011).

When normality or homogeneity of variance was not met, a nonparametric Kruskal–Wallis test was performed (adjusted *p*‐value <0.05). Pairwise comparisons, using Dunn’s test for multiple comparisons with one control, were made with *dunn.test.control* function from the *PMCMR* package (adjusted *p*‐value <0.05; Dunn, 1964).

Etoxazole toxicity was statistically analyzed between the onset and end of the population cage experiment by a general linear model with a binomial error distribution, followed by anova (using the package *stats*).

The analysis of life‐history raw data of the different lines was based on the lifetable R script (Maia, Pazianotto, Luiz, Marinho‐Prado, & Pervez, 2014). Specifically, line name, female ID, age, and number of eggs laid per female at each oviposition date, proportion of female offspring and ISS were used as input. The intrinsic rate of increase (rm) was calculated with the equation $\sum_{x=x_{0}}^{\Omega g}e^{-\mathrm{rm}}lxmx=1$ where *lx* is the proportion of females surviving to age *x* and *mx* is the mean number of female progeny per adult female at age *x*. The net reproductive rate or mean number of daughters produced per female was calculated from $\text{R0}=\sum_{x=x_{0}}^{\Omega g}lxmx$ and the mean generation time from $T=\frac{\ln(\text{R0})}{\mathrm{rm}}$. The finite rate of increase and doubling time were inferred from the equations $\text{LM}=e^{\mathrm{rm}}\;\text{and DT}=\frac{\ln2}{\mathrm{rm}}$ , respectively. Variance for the LTP parameters was estimated with Jackknife resampling method (Quenouille, 1956). As the Jackknife method is an asymptotic procedure that is sensitive to a highly skewed distribution (Maia, Luiz, & Campanhola, 2000), the symmetry of our dataset was measured with the function *skewness* from package *moments* prior to the final analysis (Sheskin, 2011). Subsequently, mean Jackknife values and their standard errors (SE) were calculated for the five LTP parameters (Meyer, Ingersoll, McDonald, & Boyce, 1986). Mean jackknife values for lines carrying mutations were then compared to the control line using Dunnett’s test (adjusted *p*‐value <0.05). Statistical analysis was conducted within the R framework [R Core Team (2014), version 3.1.2].

## 3 RESULTS

### 3.1 Development time, sex ratio, and ISS

**Figure 1:**
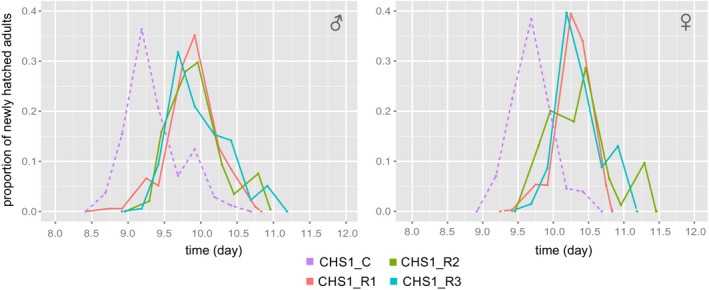
Total development time of near‐isogenic lines of *Tetranychus urticae* carrying I1017F in the *CHS1* gene and their etoxazole‐susceptible control. The experiment consisted of mites grouped in two time blocks, differing in age by 4–5 hr. Adult mites were scored in intervals of 12 hr. Total development time of males and females is presented in the left and right panel, respectively. Dashed purple line depicts adults of the susceptible EtoxR_C, while solid red, green, and blue lines depict EtoxR_R1, EtoxR_R2, and EtoxR_R3, respectively

The three lines CHS1_R1‐R3 exhibited a significantly longer median developmental time than their susceptible counterpart (CHS1_C). Both males and females of CHS1_C matured on average between 12 and 24 hr earlier than those of CHS1_R1‐R3 (Table 2, Figure 1 and Supporting Information Table S1). There was no significant difference in male and female emergence time between lines VGSC_R2, VGSC_R3 and their susceptible control (Table 2, Supporting Information Table S1). Males of the lines cytb_R1, R2, R3 did not differ from the susceptible control, while females of the cytb_R2 and R3 lines emerged significantly later than those of the control strain Wasatch (Table 2 and Supporting Information Table S1). Males and females of lines GluCl1+3_R2 and GluCl1+3_R3 had a significantly longer total developmental time when compared with the susceptible reference line (GluCl1+3_C; Table 2 and Supporting Information Table S1).

**Table 2:** Mean values ± *SE* of developmental time, immature stage survivorship (ISS), offspring sex ratio (FR), daily (FFD), and total (TF) fecundity in *Tetranychus urticae* near‐isogenic lines and the Wasatch strain

Target‐site	Line	*N* *	Developmental time ± *SE*		ISS ± *SE*	FR ± *SE*	FFD ± *SE*	TF ± *SE*
			Male	Female				
CHS1 (wt)	CHS1_C	906	**9.32 ± 0.12a**	**9.68 ± 0.08a**	0.59 ± 0.044a	0.65 ± 0.045a	6.28 ± 0.32a	91.03 ± 8.86a
CHS1 (I1017F)	CHS1_R1	710	**9.90 ± 0.06b**	**10.38 ± 0.08b**	0.63 ± 0.052a	0.67 ± 0.052a	5.52 ± 0.30a	105.41 ± 10.26a
	CHS1_R2	752	**9.96 ± 0.15b**	**10.39 ± 0.17b**	0.64 ± 0.048b	0.72 ± 0.051b	5.87 ± 0.30a	128.06 ± 10.42b
	CHS1_R3	1,132	**9.93 ± 0.09b**	**10.36 ± 0.09b**	0.58 ± 0.042a	0.65 ± 0.044a	6.57 ± 0.58a	86.06 ± 7.27a
VGSC (wt)	VGSC_C	969	10.95 ± 0.23a	11.45 ± 0.20a	0.66 ± 0.031a	0.67 ± 0.031a	8.06 ± 0.214a	101.58 ± 6.33a
VGSC (L1024V)	VGSC_R2	1,157	10.89 ± 0.16a	11.20 ± 0.18a	0.72 ± 0.02b	0.75 ± 0.026b	7.66 ± 0.204a	94.88 ± 5.69a
	VGSC_R3	1,461	11.0 ± 0.06a	11.63 ± 0.07a	0.66 ± 0.025a	0.69 ± 0.025a	8.12 ± 0.296a	93.87 ± 6.76a
Cytb (wt)	Wasatch	1,408	9.67 ± 0.38a	10.17 ± 0.38a	0.60 ± 0.037a	0.66 ± 0.037a	5.53 ± 0.28a	92.64 ± 7.85a
Cytb (P262T)	cytb_R1	1,498	9.92 ± 0.46a	10.35 ± 0.50a	0.69 ± 0.035b	0.75 ± 0.034b	6.42 ± 0.28a	113.09 ± 8.04a
	cytb_R2	1,078	10.17 ± 0.38a	10.73 ± 0.37b	0.63 ± 0.042b	0.76 ± 0.040b	4.42 ± 0.27b	71.46 ± 8.13a
	cytb_R3	1,132	9.98 ± 0.18a	10.79 ± 0.23b	0.61 ± 0.042a	0.65 ± 0.042a	5.04 ± 0.19a	94.66 ± 6.45a
GluCl1+3 (wt)	GluCl1+3_C	1,124	9.78 ± 0.09a	10.16 ± 0.06a	**0.77 ± 0.025a**	0.77 ± 0.025a	**8.32 ± 0.30a**	123.09 ± 9.64a
GluCl1+3 (G314D+G326E)	GluCl1+3_R1	1,890	10.03 ± 0.06a	10.28 ± 0.06a	**0.35 ± 0.022b**	0.43 ± 0.025b	**7.22 ± 0.34b**	93.61 ± 9.12a
	GluCl1+3_R2	1,244	10.29 ± 0.12b	10.48 ± 0.10b	**0.72 ± 0.026b**	0.76 ± 0.025a	**6.66 ± 0.36b**	105.81 ± 10.32a
	GluCl1+3_R3	1,194	10.23 ± 0.06b	10.42 ± 0.06b	**0.66 ± 0.027b**	0.67 ± 0.028b	**7.33 ± 0.344b**	120.10 ± 10.47a

Development time (d): time required for ≥50% males and females to emerge, TF: eggs/female, FFD: eggs/female/day, ISS: % females reaching adulthood, FR: % females in the progeny. Means followed by the letter “a” within a column are not significantly different from the control line (adjusted *p*‐value <0.05). *Initial number of eggs. Comparisons where all mutation‐carrying lines significantly differed from the susceptible control are indicated in bold.

Values of ISS and the ratio of female offspring for line CHS1_R2 differed significantly from line CHS1_C (Table 2, Supporting Information Table S1). VGSC_R2 had a significantly higher ISS and proportion of female surviving progeny than VGSC_C1 (Table 2, Supporting Information Table S1). The ISS and female ratio were significantly higher in cytb_R1 and R2 in comparison with the Wasatch strain (Table 2, Supporting Information Table S1). GluCl1+3_C showed significantly higher ISS values than the lines GluCl1+3_R1, R2, R3. Additionally, lines GluCl1+3_R1 and R3 were characterized by a significantly lower female ratio when compared to the control line. Line GluCl1+3_R1 exhibited an inverted sex ratio compared to the all other lines, with only 43% females in the progeny (Table 2, Supporting Information Table S1).

### 3.2 Fecundity and adult longevity

**Figure 2:**
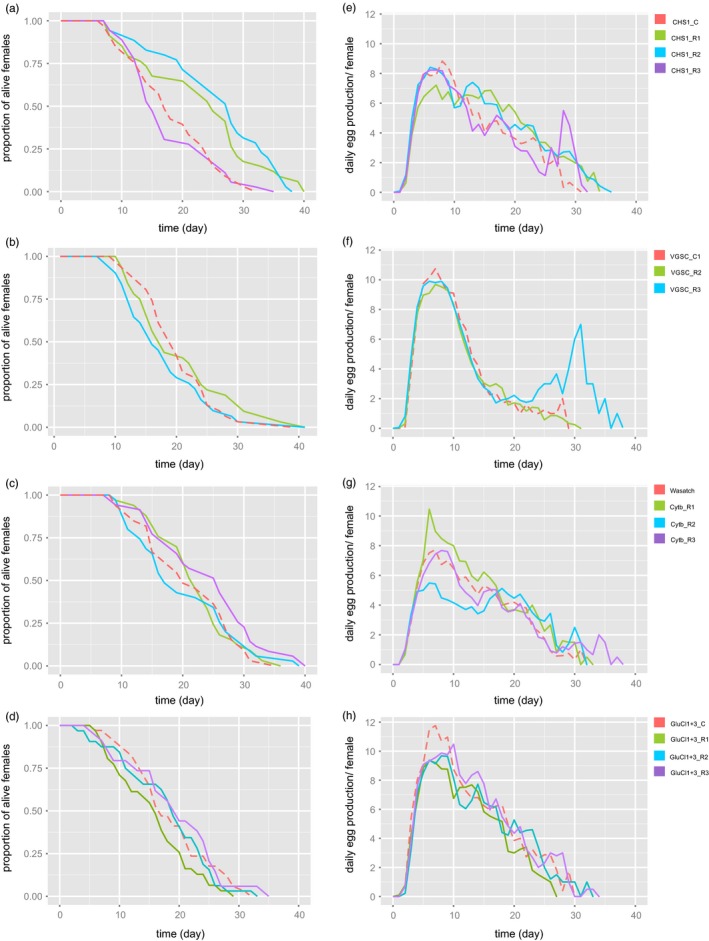
Visualization of female adult longevity and daily egg laying per *Tetranychus urticae* female. Panels a, b, c, and d present adult longevity, while panels e, f, g, and h depict the number of eggs/female/day for lines carrying I1017F in CHS1, L1024V in VGSC, P262T in cytb, and G314D and G326E in GluCl1 and 3 and their susceptible control lines, respectively. Dashed lines depict data of the susceptible control, while solid lines of different colors represent three, or in case of L1024V mutation, two lines with target‐site resistance mutation. Tables 2 and 3 represent the results of the statistical analysis on these data

Females of CHS1_R2 lived significantly longer than females of the susceptible control (CHS1_C; Figure 2a, Table 3, Supporting Information Table S1). Congenic lines VGSC_R2,3 and the susceptible control VGSC_C1 did not vary significantly in their life span (Figure 2b, Table 3, Supporting Information Table S1). Similarly, there was no difference in longevity between the three cytb_R1,2,3 lines and susceptible Wasatch (Figure 2c, Table 3, Supporting Information Table S1). Lastly, lines GluCl1+3_R1‐3 and GluCl1+3_C did not differ significantly in their life span (Figure 2d, Table 3, Supporting Information Table S1).

**Table 3:** Mean values ± *SE* of female adult longevity, duration of preoviposition (Pre‐OP), oviposition (OP), and postoviposition periods (Post‐OP) obtained for *Tetranychus urticae* near‐isogenic lines and the Wasatch strain

Target‐site	Line	*N* *	Longevity ± *SE*	Pre‐OP ± *SE*	OP ± *SE*	Post‐OP ± *SE*
CHS1 (wt)	CHS1_C	33	18.182 ± 1.22a	1.34 ± 0.05a	14.03 ± 1.19a	1.64 ± 0.43a
CHS1 (I1017F)	CHS1_R1	34	23.147 ± 1.68a	1.4 ± 0.05a	18.32 ± 1.41a	1.12 ± 0.30a
	CHS1_R2	34	25.657 ± 1.49b	1.2 ± 0.06a	21.44 ± 1.36b	2.15 ± 0.39a
	CHS1_R3	35	17.389 ± 1.17a	1.21 ± 0.06a	14.2 ± 1.07a	1.00 ± 0.35a
VGSC (wt)	VGSC_C1	31	20.16 ± 1.07a	1.39 ± 0.05a	12.77 ± 0.87a	4.45 ± 0.78a
VGSC (L1024V)	VGSC_R2	32	20.50 ± 1.43a	1.36 ± 0.05a	12.84 ± 1.01a	4.81 ± 1.03a
	VGSC_R3	31	17.90 ± 1.31a	1.33 ± 0.07a	11.84 ± 1.07a	3.32 ± 0.63a
Cytb (wt)	Wasatch	33	21.12 ± 1.25a	1.59 ± 0.09a	16.45 ± 1.12a	1.82 ± 0.24a
Cytb (P262T)	cytb_R1	33	21.97 ± 1.1a	1.41 ± 0.09a	17.24 ± 0.97a	2.03 ± 0.43a
	cytb_R2	35	20.20 ± 1.43a	1.53 ± 0.09a	15.29 ± 1.34a	2.26 ± 0.45a
	cytb_R3	35	24.03 ± 1.41a	1.41 ± 0.07a	18.8 ± 1.19a	1.66 ± 0.41a
GluCl1+3 (wt)	GluCl1+3_C	34	19.53 ± 1.16a	**1.33 ± 0.05a**	14.74 ± 1.16a	2.26 ± 0.39a
GluCl1+3 (G314D+G326E)	GluCl1+3_R1	31	16.71 ± 1.14a	**1.66 ± 0.07b**	12.52 ± 11a	1.32 ± 0.32a
	GluCl1+3_R2	32	19.00 ± 1.32a	**1.54 ± 0.07b**	15.25 ± 1.36a	0.75 ± 0.22b
	GluCl1+3_R3	34	19.12 ± 1.35a	**1.56 ± 0.08b**	15.44 ± 1.29a	1.76 ± 0.38a

Time is expressed in days. Means followed by the letter “a” within a column are not significantly different from the control line (adjusted *p*‐value <0.05). *Number of females. Comparisons where all mutation‐carrying lines significantly differed from the susceptible control are indicated in bold.

There was no significant difference between CHS1_C and CHS1_R1‐3 in daily egg laying per female. However, line CHS1_R2 showed a significantly higher total fecundity per female compared to CHS1_C (Figure 2e, Table 2, Supporting Information Table S1). Lines VGSC_C1 and VGSC_R2,3 did not differ significantly in their daily and total fecundity (Figure 2f, Table 2, Supporting Information Table S1). The cytb_R2 line exhibited a lower daily egg laying when compared to the Wasatch control. There was no significant difference between Wasatch and the cytb_R1‐R3 in total fecundity per female (Figure 2g, Table 2, Supporting Information Table S1). The control line GluCl1+3_C displayed the highest and a significantly different daily fecundity compared to the three GluCl1+3_R1‐3 lines, while values for the mean total fecundity did not differ significantly between control and the lines with mutation (Figure 2h, Table 2, Supporting Information Table S1).

Of note, line cytb_R2 displayed an unusual egg laying pattern, lacking the characteristic oviposition peak, but exhibited a prolonged plateau that extended over a substantial proportion of the oviposition period (Figure 2g). This may explain the observed low mean values for total and daily fecundit y (Table 2). The plots of daily egg laying of lines CHS1_R3 and VGSC_R3 displayed an unusual shape with an additional oviposition peak around the 30th day after eclosion. In each experiment, the peak resulted from a prolonged oviposition period of one female mite (Figure 2e,f).

### 3.3 Preoviposition, oviposition and postoviposition periods

Lines CHS1_R1,R2,R3 did not differ significantly in the length of the preoviposition period compared to CHS1_C (Table 3, Supporting Information Table S1). CHS1_C had significantly shorter oviposition periods compared to CHS1_R2. Lines CHS1_R1,R2,R3 and CHS1_C did not differ significantly in the duration of the postoviposition period (Table 3, Supporting Information Table S1). Lines VGSC_R2, 3 and VGSC_C1 did not differ significantly in the length of preoviposition, oviposition or postoviposition periods, neither did the lines cytb_R1,2,3 in comparison with Wasatch (Table 3, Supporting Information Table S1). GluCl1+3_C had a significantly shorter preoviposition period than lines GluCl1+3_R1, R2, R3. The lines did not differ significantly in the length of the oviposition period, but GluCl1+3_R2 had significantly shorter postoviposition period compared to GluCl1+3_C (Table 3, Supporting Information Table S1).

### 3.4 Fertility life table parameters

Mean Jackknife values of LTP parameters for lines with mutations in CHS1, VGSC, cytb, and GluCl channels and their respective control lines are summarized in Table 4. With the exception of line VGSC_R2 (1.19), results of the skewness test indicated that the data were fairly symmetrically distributed (rule of a thumb −0.5 ≤ *x* ≤ 0.5) or moderately skewed (−1 ≤ x ≤ 1), justifying the use of Jackknife resampling method (Supporting Information Table S2, Figures [Link], [Link], [Link], [Link]).

**Table 4:** Jackknife estimates ± *SE* of five LTP parameters obtained for near‐isogenic lines of *Tetranychus urticae* and Wasatch

Target‐site	Line	*N* *	R0 ± *SE*	*T* ± *SE*	DT ± *SE*	rm ± *SE*	LM ± *SE*
CHS1 (wt)	CHS1_C	33	35.31 ± 3.44a	**17.96 ± 0.30a**	3.49 ± 0.06a	0.199 ± 0.003a	1.220 ± 0.004a
CHS1 (I1017F)	CHS1_R1	34	44.23 ± 4.31a	**20.15 ± 0.33b**	3.68 ± 0.07b	0.188 ± 0.004b	1.207 ± 0.004b
	CHS1_R2	34	58.83 ± 4.79b	**19.56 ± 0.30b**	3.32 ± 0.05a	0.208 ± 0.003a	1.232 ± 0.004a
	CHS1_R3	35	32.37 ± 2.74a	**18.28 ± 0.33b**	3.64 ± 0.06a	0.190 ± 0.003a	1.210 ± 0.004a
VGSC (wt)	VGSC_C1	31	46.32 ± 3.03a	19.41 ± 0.33a	3.505 ± 0.05a	0.198 ± 0.003a	1.219 ± 0.003a
VGSC (L1024V)	VGSC_R2	32	51.33 ± 3.08a	19.35 ± 0.21a	3.403 ± 0.02a	0.204 ± 0.001a	1.226 ± 0.002a
	VGSC_R3	31	42.99 ± 3.10a	19.38 ± 0.33a	3.568 ± 0.04a	0.194 ± 0.002a	1.214 ± 0.003a
Cytb (wt)	Wasatch	33	36.93 ± 3.13a	19.52 ± 0.29a	3.74 ± 0.07a	0.185 ± 0.004a	1.203 ± 0.004a
Cytb (P262T)	cytb_R1	33	58.30 ± 4.15b	19.48 ± 0.25a	3.32 ± 0.05b	0.209 ± 0.003b	1.232 ± 0.004b
	cytb_R2	35	34.23 ± 3.89a	20.05 ± 0.4a	3.92 ± 0.09a	0.177 ± 0.004a	1.193 ± 0.005a
	cytb_R3	35	38.15 ± 2.54a	20.06 ± 0.27a	3.82 ± 0.07a	0.182 ± 0.003a	1.199 ± 0.004a
GluCl1+3 (wt)	GluCl1+3_C	34	**73.40 ± 5.75a**	**18.44 ± 0.22a**	**2.97 ± 0.03a**	**0.233 ± 0.003a**	**1.262 ± 0.003a**
GluCl1+3 (G314D+G326E)	GluCl1+3_R1	31	**13.99 ± 1.36b**	**19.18 ± 0.26b**	**5.03 ± 0.15b**	**0.138 ± 0.004b**	**1.148 ± 0.005b**
	GluCl1+3_R2	32	**57.61 ± 5.53b**	**19.53 ± 0.28b**	**3.33 ± 0.07b**	**0.208 ± 0.004b**	**1.231 ± 0.005b**
	GluCl1+3_R3	34	**52.78 ± 4.60b**	**19.65 ± 0.23b**	**3.43 ± 0.06b**	**0.202 ± 0.003b**	**1.224 ± 0.004b**

Means followed by the letter “a” within a column are not significantly different from the control line (adjusted *p*‐value <0.05). *Number of females. Comparisons where all mutation‐carrying lines significantly differed from the susceptible control are indicated in bold.

Line CHS_R1 was found to have significantly smaller values of rm and LM and consequently significantly longer doubling time (DT) than CHS_C. Line CHS1_R2 was characterized with a higher net reproductive rate (R0) compared to CHS1_C. The control line had a significantly shorter generation time (T) when compared to CHS1_R1,R2,R3 (Table 4).

The VGSC_R2,3 lines did not differ significantly from the susceptible VGSC_C1 congenic line in any of the five LTP parameters.

R0, DT, rm, LM, and T did not differ between control line Wasatch and cytb_R2 and R3. Line Cytb_R1 showed the highest R0, rm, and LM and shortest DT. Mean generation time was the only parameter that did not differ in a significant way between all four lines (Table 4).

All LTP parameters were significantly different between GluCl1+3_R1‐3 and their susceptible congenic line (Table 4). The control line had significantly higher values of rm, LM and R0 and consequently lower values for T and DT. As mentioned above, line GluCl1+3_R1 was characterized with an inverted sex ratio and consequently was repeatedly inferior to the remaining lines for all LTP parameters tested, with an exception of mean generation time (Table 4).

### 3.5 Temporal dynamics of etoxazole resistance

**Figure 3:**
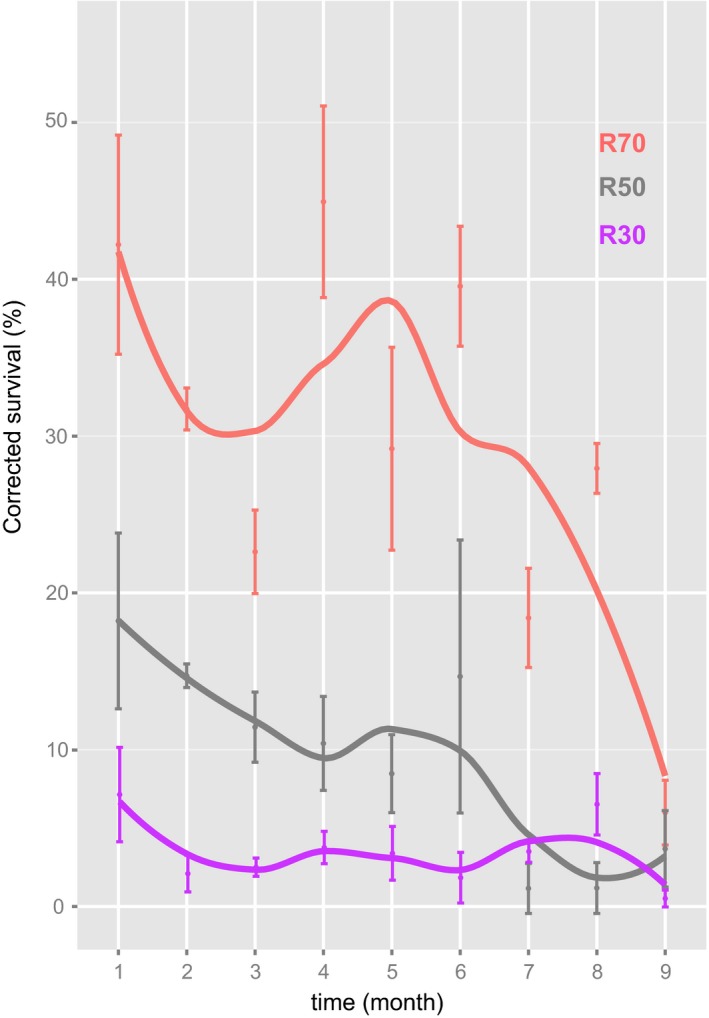
Visualization of the temporal dynamics of etoxazole resistance in *Tetranychus urticae*. Nonparametric loess curves were fitted to the data using the *lowess* function in R. Red color depicts population R70, gray R50 and purple R30, that is, populations with 70%, 50% and 30% of resistant homozygous females in the starting population, respectively

Our experimental data revealed that, after approximately three generations (i.e., 1 month at 28°C), the percentage of mites that survived etoxazole exposure and thus were homozygous for the CHS1 resistance mutation was 42%, 18%, and 7% for the R70, R50, and R30 populations, respectively. These values were below the frequencies expected under Hardy–Weinberg equilibrium (49%, 25%, and 9%, respectively). Following 8 months of population propagation, the frequencies of resistant homozygous individuals decreased to 6.0%, 4.1%, and 0.5% for R70, R50, and R30, respectively (Figure 3). For each population, the difference in the resistance level was significant between the onset and end of the cage experiment (R30: Chisq_1_ = 6.91, *p*‐value <0.05; R50: Chisq_1_ = 37.75, *p*‐value <0.05; R70: Chisq_1_ = 14.47, *p*‐value <0.05).

## 4 DISCUSSION

Mutations that underlie target‐site resistance can carry a fitness disadvantage in an insecticide/acaricide‐free environment (Crow, 1957; Fisher, 1999). In contrast to target‐site resistance mutations that have been present within pest populations as natural balanced polymorphisms prior to the exposure of the insecticide/acaricide, de novo formed resistant alleles are expected to generate a high fitness disadvantage in resistant arthropods, compared to their susceptible conspecifics (ffrench‐Constant, 2007; ffrench‐Constant & Bass, 2017; Fisher, 1999). In light of this theory, the study of the potential biological weaknesses that are associated with acaricide/insecticide resistance in populations is of high importance in the context of Insecticide Resistance Management (IRM; Crow, 1957; Georghiou & Taylor, 1977). The origin and history of the nucleotide polymorphisms associated with resistance remains largely unknown (but see Gould et al., 1997; Hartley et al., 2006), which lowers the reliability of a priori predictions of potential fitness costs. In this study, we took advantage of a collection of near‐isogenic lines to quantify potential pleiotropic fitness effects of five key mutations associated with resistance in *T. urticae*. Three target‐site resistance mutations, I1017F in CHS1 and the co‐occurring G314D and G326E mutations in GluCl1 and GluCl3, were shown to significantly and consistently alter certain fitness parameters in *T. urticae* (Table 2). The other two mutations, P262T in cytb and L1024V in VGSC, did not induce a consistent change in any fitness parameter that was analyzed in our study.

Chitin synthase, the rate limiting enzyme in the chitin biosynthesis pathway of arthropods, is targeted by a number of classes of insecticides/acaricides referred to as chitin synthesis inhibitors (IRAC 15, e.g., benzylurea; IRAC 16, buprofezin) and mite growth inhibitors (IRAC 10, clofentezine, hexythiazox, and etoxazole; Demaeght et al., 2014; Douris et al., 2016; Van Leeuwen et al., 2012). Resistance to mite growth inhibitors and chitin synthase inhibitors has been attributed to the I1017F mutation in chitin synthase (Demaeght et al., 2014; Douris et al., 2016; Grigoraki et al., 2017; Riga et al., 2017; Van Leeuwen et al., 2012). As the I1017F mutation is located in a highly conserved region of the *CHS1* gene (Douris et al., 2016; Grigoraki et al., 2017; Suzuki, Shiotsuki, Jouraku, Miura, & Minakuchi, 2017; Yamamoto, Yoneda, Hatano, & Asada, 1995)*,* this substitution could impair the enzymatic function. In the current study, the impact of I1017F on spider mite fitness was quantified by analyzing single‐generation life‐history traits, fertility life table parameters of individual near‐isogenic lines as well as by an interline competition experiment. Results showed that the total development and mean generation time were significantly different between the strains carrying the I1017F mutation in relation to the control strain (Figure 1, Tables 2 and 4). The analysis of the temporal dynamics of etoxazole resistance in mixed population experiment (Figure 3) showed that in all three populations (R30, R50, and R70), the frequency of resistant homozygotes dropped below 10% in a period of 8 months (Figure 3). Interestingly, the expression of *T. urticae CHS1* is the highest in the obligate chrysalis stage wherein the mite molts to complete its development (Van Leeuwen et al., 2012). Mite development time could thus be affected by the I1017F mutation by inefficient molting and a longer chrysalis stage. In support of this theory, *Plutella xylostella* strains that carry the I1042M mutation, which is located at the homologous position to the I1017F *T. urticae* mutation, had a significantly longer development time at 20 and 30°C, compared to strains lacking the mutation (Steinbach, Moritz, & Nauen, 2017). In addition to a shorter development time, the I1042M mutation is also associated with a lower fecundity in the resistant vs susceptible *P. xylostella* strains (Steinbach et al., 2017). It should however be noted that CrispR/Cas9 genome edited *D. melanogaster* flies that bear the *T. urticae* I1017F or the *P. xylostella* I1042M mutation do not exhibit a significant difference in time until eclosion, adult survival, or average daily fecundity, compared to wild‐type flies with an isogenic background (Douris et al., 2016). This indicates that the I1017F/M mutations (*T. urticae* numbering) may not have or may have different pleiotropic effects across arthropod species.

Ilias, Vontas, and Tsagkarakou (2014) found that the I1017F mutation is relatively widespread and often present in a homozygous state in field and laboratory *T. urticae* populations originating from a wide geographic range (Europe, Asia and Africa). Our results, based on the two lines of evidence that identified a significant fitness cost associated with the I1017F mutation in the current study, may at first appear contradictory to the findings of Ilias et al. (2014). However, the high number of resistant homozygotes in Ilias et al. (2014) is very likely biased by sampling mite populations from ornamental flowers. These ornamentals are exposed to a heavy use of acaricides, a pest management strategy that can result in the maintenance of the I1017F mutation in homozygosity within these mite populations, as several mite growth inhibitors are still frequently used worldwide (Demaeght et al., 2014). Second, we may have overlooked a potential fitness advantage of the *CHS1* target‐site mutation, or of a physically linked locus, that did not surface under the stable laboratory conditions of our experiments, but helps field *T. urticae* populations survive spatial and temporal heterogeneous environmental conditions.

As the I1017F mutation in *CHS1* significantly diminished the fitness of our experimental *T. urticae* populations, pesticide management strategies could exploit the selective advantage of etoxazole‐susceptible spider mites by creating unsprayed refugia where these can propagate. Indeed, previous studies suggest that a high‐dose‐refugee strategy is particularly effective in reverting resistance when that is functionally recessive, as etoxazole resistance by mutations in *CHS1* (Alphey, Coleman, Bonsall, & Alphey, 2008; Carrière & Tabashnik, 2001; Huang, Andow, & Buschman, 2011; Tabashnik, Gould, & Carrière, 2004). Additionally, creating sufficiently lengthy time intervals between treatments can allow etoxazole‐susceptible mites to reproduce and re‐establish susceptibility in *T. urticae* field populations (Leeper, Roush, & Reynolds, 1986). It has to be noted however that our population cage experiment was performed at a high temperature (28°C). At this temperature, the generation turnover in *T. urticae* and other spider mite species is very fast. Hence, the observed significant loss of resistance in a duration of 8 months may reflect an upper limit for what might be expected under suboptimal field conditions. Therefore for the optimal management of etoxazole resistance in the field, it may be necessary to extend the intervals between the spray treatments beyond the recommended, one treatment per cropping season (Borneo, 2007).

Abamectin and milbemectin primarily target GluCl channels. Reports of abamectin/milbemectin field resistance remain relatively scarce for *T. urticae*, despite the heavy use of both compounds against spider mite infestations for more than 30 years (Campos, Dybas, & Krupa, 1995; Campos, Krupa, & Dybas, 1996; Kwon, Lee, Ahn, & Lee, 2014; Stumpf & Nauen, 2002; Van Leeuwen et al., 2010). Abamectin resistance is known to evolve through different mechanisms and can include target‐site and/or biochemical/metabolic resistance (Dermauw et al., 2012; Kwon, Yoon, et al., 2010; Pavlidi et al., 2015; Riga et al., 2014, 2017). Previous studies have however shown that target‐site resistance is only of minor importance for the occasionally encountered high resistance levels in field populations (Riga et al., 2017).

In the current study, the near‐isogenic GluCl1+3_R1‐3 lines, that carry both the G314D and G326E mutations, exhibited significantly lower ISS and longer preoviposition period as well as lower daily fecundity/female, compared to the GluCl1+3_C control line (Table 2). Differences in single life‐history traits were reflected in LTPs; the three resistant lines had significantly lower values of R0, rm and LM and consequently higher T and DT compared to the abamectin‐susceptible control (Table 4).

The closely located A309V mutation (*T. urticae* numbering) at the N‐terminus of the third transmembrane helix (TM3) of the GluCl channel is strongly associated with abamectin resistance in *P. xylostella* (Wang & Wu, 2014). In parallel to our results, Wang and Wu (2014) conclude that abamectin resistance carries significant fitness costs in *P. xylostella* after backcrossing the abamectin‐resistant Roth‐Abm strain carrying the A309V mutation into the parental abamectin‐susceptible Roth strain. A subsequent study showed that the frequency of the resistance allele decreased from 94.7% to 9.6% in populations after 20 generations of no abamectin exposure, further confirming a pleiotropic effect of the A309V mutation (Wang et al., 2016). Although the *T. urticae* and *Plutella* mutations are not located at an identical position, they are situated at the *N*‐terminus of TM3. The TM2, TM3, and TM2–TM3 linker regions have been previously shown to be critical for the function of the ligand‐gated chloride channels (Kane et al., 2000; Lynagh, Webb, Dixon, Cromer, & Lynch, 2011; Wang & Wu, 2014).

A combination of the two GluCl mutations does not confer high resistance levels to abamectin in *T. urticae* (Riga et al., 2017) and may thus act as a resistance limiting factor, maintaining a high efficiency of abamectin, regardless of its long history of use (Pavlidi et al., 2015; Riga et al., 2014). This scenario finds its support in the worldwide survey by Ilias et al. (2014), where the combination of the two mutations was only present in two field collections (i.e., 0.06% of all samples), both originating from abamectin‐treated greenhouses (Ilias et al., 2014). A recent electrophysiological study on *T. urticae* GluCl3 has shown that homomeric wild‐type and G326E GluCl3 channel does not differ in performance in the absence of acaricides when expressed in *Xenopus laevis* oocytes (Mermans et al., 2017). Our results now raise the question whether different combinations of target‐site mutations affect channel performance and whether hetero‐ or homomeric GluCl channels are formed in vivo in *T. urticae*. As G326E occurs relatively frequently in worldwide isolates of *T. urticae* (21% of all samples)*,* compared to the G314D mutation, or the combination of both mutations (Ilias et al., 2014); thus, the G326E mutation alone may not impose significant fitness costs.

The G314D and G326E mutations in GluCls had a significant effect on the LTP values of our experimental *T. urticae* populations. Although we did not perform a cage experiment where we placed the resistant mites in direct competition with their susceptible counterparts, our results strongly suggest that a combination of two mutations confers a pronounced negative effect on *T. urticae* fitness. When the pesticide resistance‐related fitness cost is of such a large magnitude, the use of two unrelated MoA pesticides in rotation as a pest management strategy would allow to completely restore abamectin/milbemectin susceptibility (Overmeer, Van Zon, & Helle, 1975).

There were no consistent significant differences observed in the LHTs, nor in LTPs between the control line and the pyrethroid‐resistant lines that carry the L1024V mutation within the *T. urticea VGSC* gene. In contrast, using near‐isogenic lines, the kdr and/or super‐kdr mutations in VGSCs of various insect species have previously been shown to induce changes in life‐history parameters [for instance oviposition in *A. aegypti* (Brito et al., 2013) and survival to adulthood in *C. quinquefasciatus* (Berticat et al., 2008)]. Moreover, these mutations also appear to affect nerve functioning and behavior in insect species. For example, a pyrethroid‐resistant strain of *Musca domestica* responds differently to changes in temperature (Foster et al., 2003) and pyrethroid resistance in *A. gambiae* and *A. aegypti* is associated with a lowered ability of host seeking and an increased locomotor activity, respectively (Brito et al., 2013; Diop et al., 2015). It is therefore possible that we have missed a potential pleiotropic effect of the L1024V mutation on the behavior of *T. urticae*.

The P262T mutation of the *T. urticae cytb* gene also did not alter any of the quantified fitness components in this study. Previous studies that focused on genetically related (but not isogenic) susceptible and bifenazate‐resistant strains show that the nearby positioned S141F + G126S mutations in the cd1 helices of the *cytb* gene also do not induce significant differences in individual life‐history traits and intrinsic rate of increase (Van Leeuwen et al., 2008; Van Pottelberge, 2008). Interestingly however, Van Pottelberge (2008) found that when the same resistant and susceptible strains are placed in direct competition, bifenazate‐resistant mites are less competitive in the absence of selection pressure compared to susceptible mites, raising the question how our isogenic lines would compete with one another.

In this study, we performed a comprehensive analysis of the potential fitness cost associated with key target‐site resistance mutations in *T. urticae*. The target‐site mutations in *CHS1* and *GluCl* channels conferring resistance to chitin synthesis inhibitors and avermectins, respectively, impose a significant fitness cost in *T. urticae*. This finding may be of crucial importance for integrated pest management (IPM) strategies. The apparent absence of a significant fitness cost in *T. urticae* for the target‐site mutations in *cytb* and *VGSC*, conferring resistance to bifenazate and pyrethroids, respectively, has to be approached with some caution, as it remains possible that subtle pleiotropic effects only appear under specific conditions [such as food shortage, high rate of migration and at certain population sizes or densities (ffrench‐Constant & Bass, 2017; McKenzie, 1996)].

## DATA AVAILABILITY

Data available from the Dryad Digital Repository: https://doi.org/10.5061/dryad.32ht688.

## CONFLICT OF INTEREST

The authors declare no conflict of interest related to this manuscript.
