# Supplementary figures and images for: Fitness costs of key point mutations that underlie acaricide target‐site resistance in the two‐spotted spider mite Tetranychus urticae
Source: Evol Appl. 2018 May 20;11(9):1540–53. doi: 10.1111/eva.12643 (PMC6183448; doi:10.1111/eva.12643)

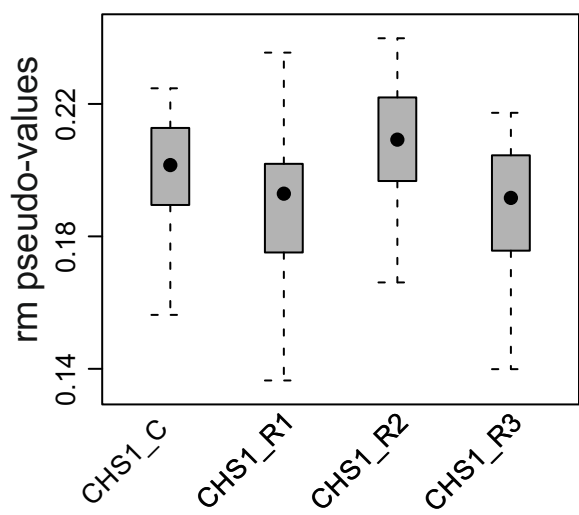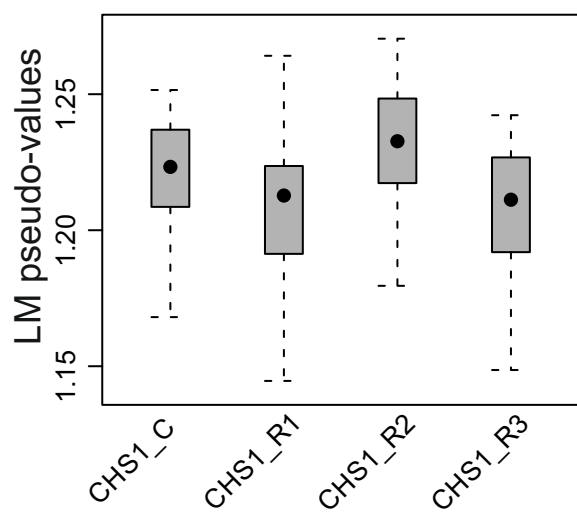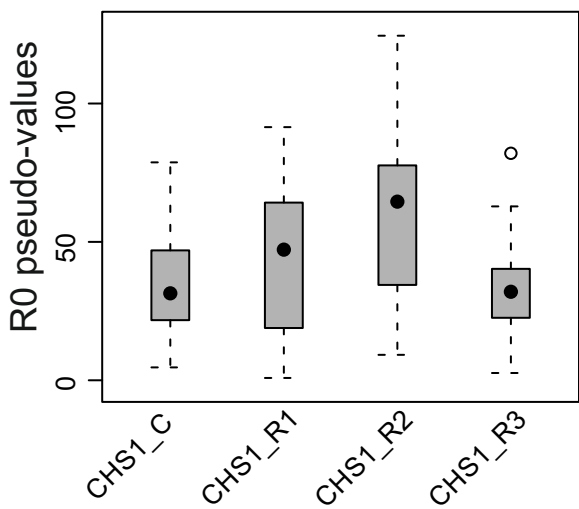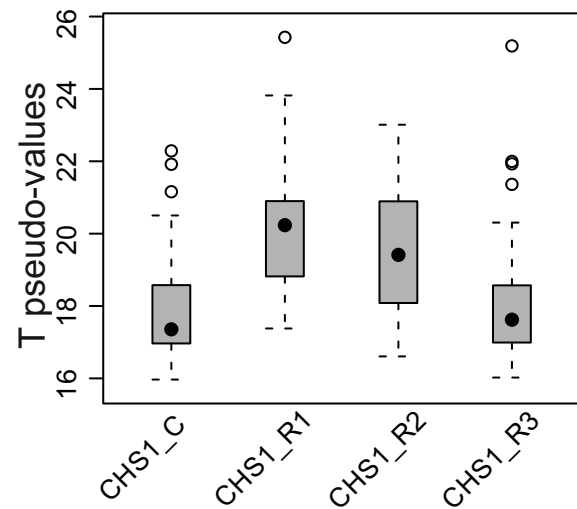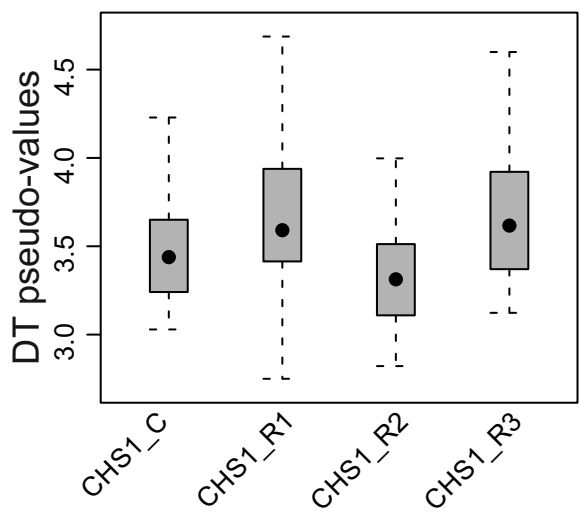

Supplement: Supplementary file 1 [file EVA-11-1540-s001.pdf]

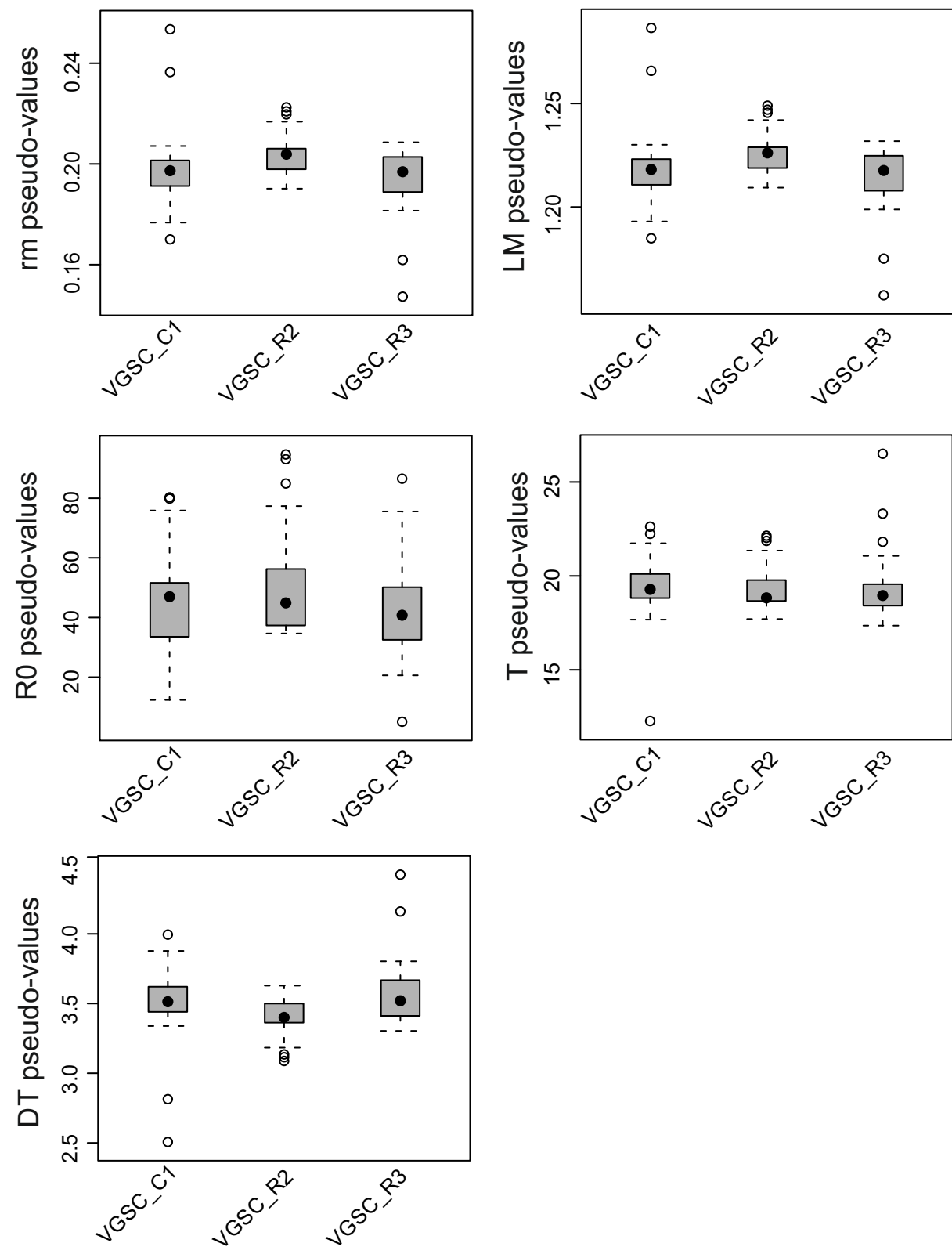

Supplement: Supplementary file 2 [file EVA-11-1540-s002.pdf]

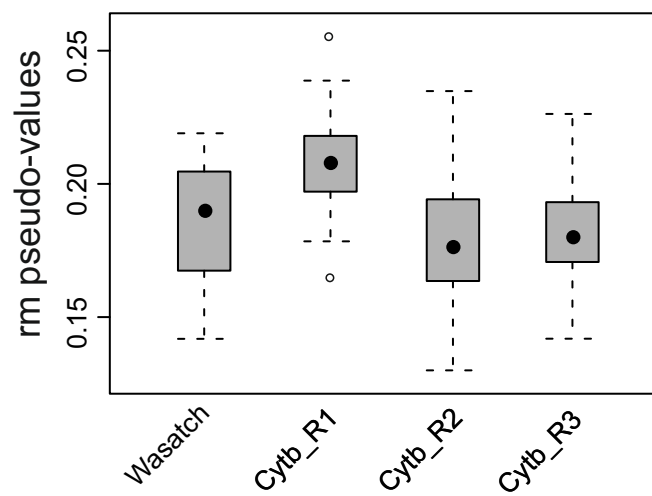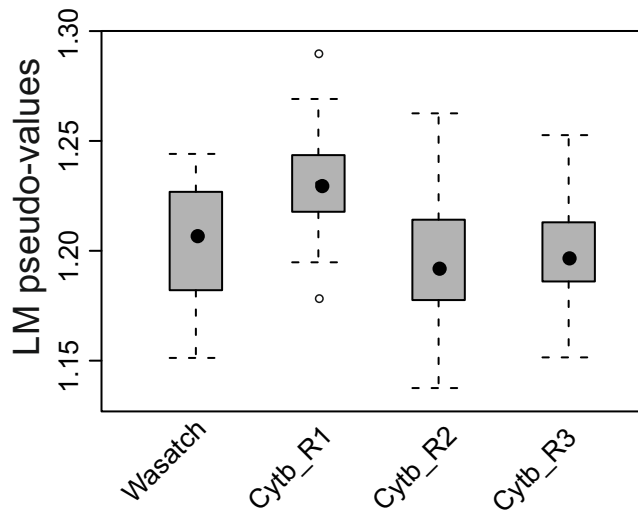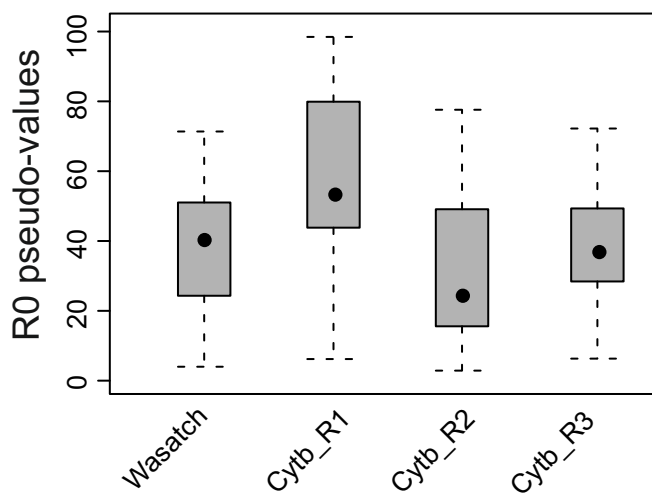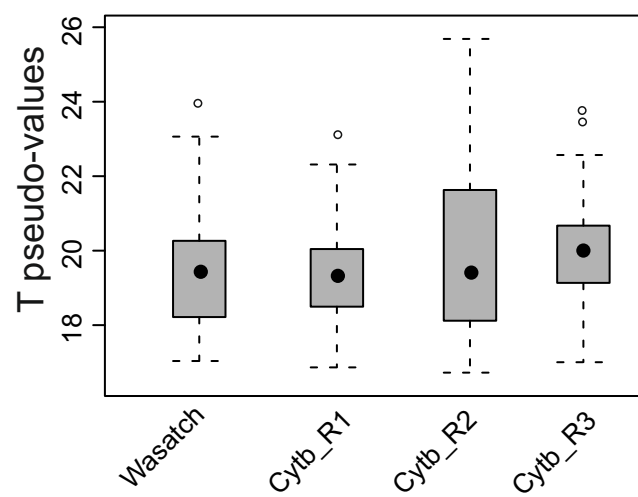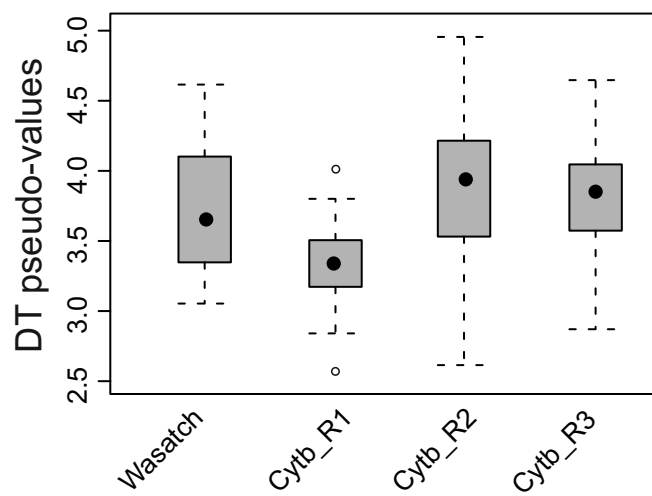

Supplement: Supplementary file 3 [file EVA-11-1540-s003.pdf]

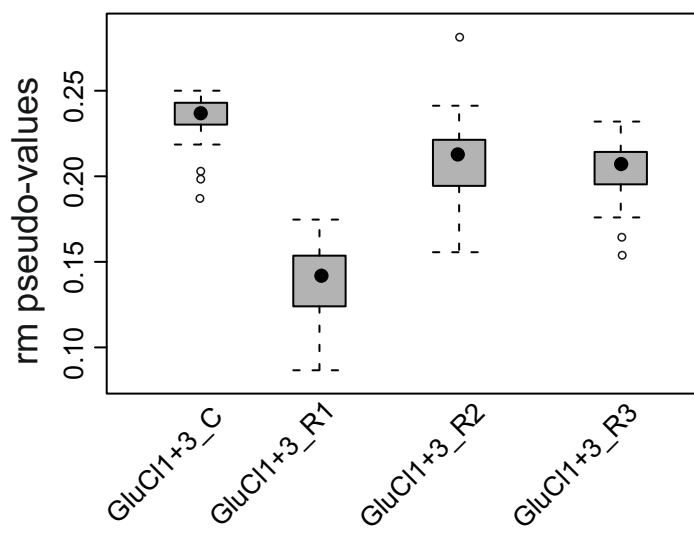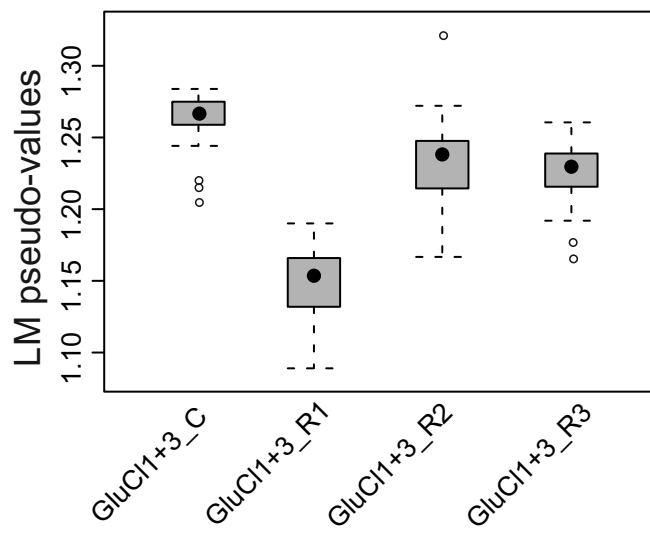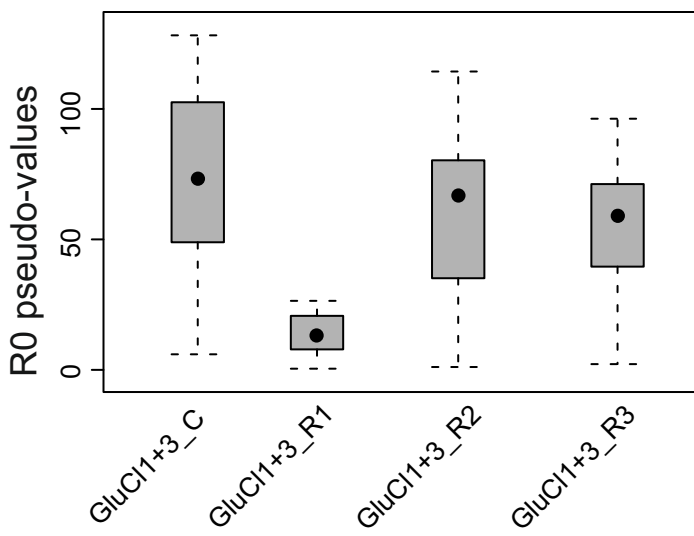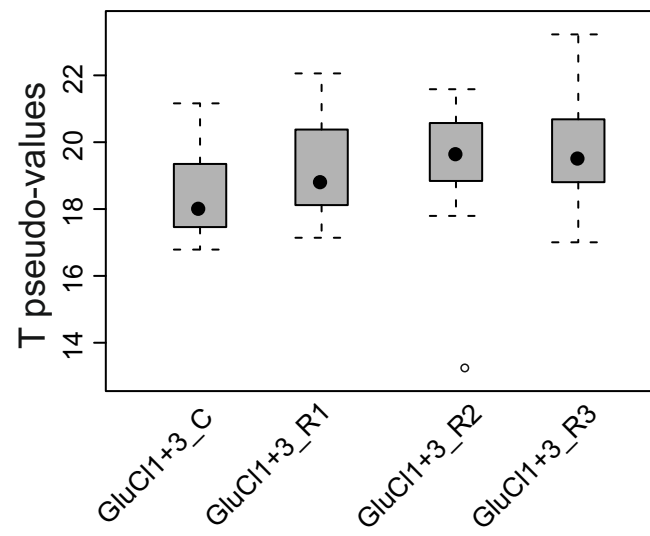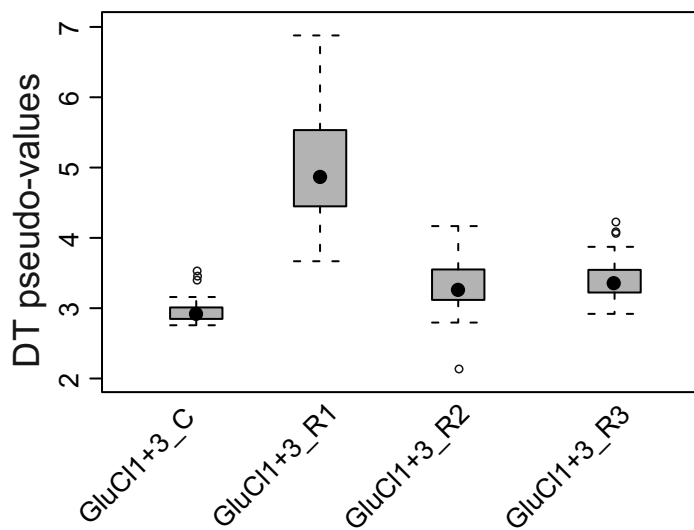

Supplement: Supplementary file 4 [file EVA-11-1540-s004.pdf]
