# Supplementary material for: Fitness costs of key point mutations that underlie acaricide target‐site resistance in the two‐spotted spider mite Tetranychus urticae
Source: Evol Appl. 2018 May 20;11(9):1540–53. doi: 10.1111/eva.12643 (PMC6183448; doi:10.1111/eva.12643)
Supplement: Supplementary file 5 [file EVA-11-1540-s005.docx]

| parameter | sex | CHS1_R1,2,3 | Cytb_R1,2,3 | VGSC_R2,3 | GluCl1+3_R1, R2, R3 |
| --- | --- | --- | --- | --- | --- |
| developmental time | male | F_3_= 7.64, **p-value < 0.05** | Chisq_3_=6.00, **p-value > 0.05** | F_2_= 0.19, **p-value > 0.05** | F_3_= 7.49, **p-value < 0.05** |
|  | female | F_3_=9.99, **p-value < 0.05** | Chisq_3_= 10.84**, p-value < 0.05** | *Chisq_2_= 1.68, **p-value < 0.05** | Chisq_3_= 14.37, **p-value < 0.05** |
| ISS | | Chisq_3_=7.83, **p-value < 0.05** | Chisq_3_= 51.24, **p-value < 0.05** | Chisq_2_= 14.65, **p-value < 0.05** | Chisq_3_= 15.8, **p-value<0.05** |
| offspring sex ratio | | Chisq_3_= 13.13, **p-value < 0.05** | Chisq_3_=71.479, **p-value < 0.05** | Chisq_2_=21.02, **p-value < 0.05** | Chisq_3_= 10.09, **p-value < 0.05** |
| daily fecundity | | Chisq_3_= 2.13, **p-value > 0.05** | Chisq_3_= 26.87, **p-value < 0.05** | Chisq_2_= 3.48, **p-value > 0.05** | Chisq_3_= 18.89, **p-value < 0.05** |
| total fecundity | | Chisq_3_= 10.35, **p-value <0.05** | *Chisq_3_= 13.12, **p-value < 0.05** | Chisq_3_= 5.87, **p-value > 0.05** | Chisq3= 5.87, **p-value >0.05** |
| longevity | | Chisq_3_=19.29, **p-value < 0.05** | Chisq_3_= 4.66,  **p-value > 0.05** | Chisq_2_= 3.23,  **p-value > 0.05** | F_3_=0.99**, p-value > 0.05** |
| pre-oviposition | | *Chisq_3_= 8.30, **p-value < 0.05** | Chisq_3_= 1.59, **p-value > 0.05** | Chisq_2_= 0.08, **p-value > 0.05** | Chisq_3_= 12.12, **p-value < 0.05** |
| oviposition | | Chisq_3_=19.49, **p-value < 0.05** | F_3_= 1.63, **p-value > 0.05** | Chisq_2_= 1.86, **p-value > 0.05** | Chisq_3_= 3.71, **p-value > 0.05** |
| post-oviposition | | Chisq_3_= 5.55, **p-value > 0.05** | Chisq_3_= 2.23, **p-value > 0.05** | Chisq_2_= 1.23, **p-value > 0.05** | Chisq_3_= 10.88, **p-value < 0.05** |

Table S1. Results of the analysis of variance

* There is a significant difference among the mutation carrying lines but not between the susceptible control and the mutation carrying lines (see table 2 and table 3)
