# Supplementary material for: Fitness costs of key point mutations that underlie acaricide target‐site resistance in the two‐spotted spider mite Tetranychus urticae
Source: Evol Appl. 2018 May 20;11(9):1540–53. doi: 10.1111/eva.12643 (PMC6183448; doi:10.1111/eva.12643)
Supplement: Supplementary file 6 [file EVA-11-1540-s006.docx]

| Line | Skewness |
| --- | --- |
| CHS1_C | 0.44 |
| CHS1_R1 | -0.11 |
| CHS1_R2 | 0.06 |
| CHS1_R3 | 0.78 |
| VGSC_C1 | 0.25 |
| VGSC_R2 | 1.19 |
| VGSC_R3 | 0.55 |
| Wasatch | -0.18 |
| Cytb_R1 | -0.14 |
| Cytb_R2 | 0.47 |
| Cytb_R3 | -0.03 |
| GluCl1+3_C | -0.08 |
| GluCl1+3_R1 | -0.01 |
| GluCl1+3_R2 | -0.32 |
| GluCl1+3_R3 | -0.48 |

Table S2. Measure of skewness of the data distribution (total fecundity/ female within a line) for the *Tetranychus urticae* lines used in this study.
